# Supplementary material for: Analysis of technical efficiency of irrigated onion (Allium cepa L.) production in North Gondar Zone of amhara regional state, Ethiopia
Source: PLoS One. 2022 Oct 13;17(10):e0275177. doi: 10.1371/journal.pone.0275177 (PMC9562163; doi:10.1371/journal.pone.0275177)
Supplement: S1 File — (DOCX) [file pone.0275177.s002.docx]

**University of Gondar**

**College of Agriculture and Rural Transformation**

**Department of Agricultural Economics**

**Interview Schedule to Analysis of Technical Efficiency of Irrigated Onion (*Allium cepa* L*.*) Production in North Gondar Zone of Amhara Regional State, Ethiopia**

**Dear Respondents:** This interview schedule is prepared to undertake a study on the ***“*Analysis of Technical Efficiency of Irrigated Onion (*Allium cepa* L*.*) Production in North Gondar Zone of Amhara Regional State, Ethiopia”**. The information that you are going to provide based on specific questions will be used only for the research purpose and will not be disclosed for any third party. You are kindly requested to participate in filling the questions.

1. Age _____________ (years)
2. Number of years of formal education of the head of the household? __________
3. For how many years did you cultivate onion? _____ Years.
4. Family members of the household

| **Sex** | **Age Category** | | | | | **Remark** |
| --- | --- | --- | --- | --- | --- | --- |
|  | **<10** | **10-13** | **14-16** | **17-50** | **>50** |  |
| Male |  |  |  |  |  |  |
| Female |  |  |  |  |  |  |

**5. Total number of livestock holding by the household head**

| S/N | Type of Herd | Total number |
| --- | --- | --- |
| 1 | Cow |  |
| 2 | Oxen |  |
| 3 | Heifer |  |
| 4 | Calves |  |
| 5 | Sheep |  |
| 6 | Steers |  |
| 7 | Goats |  |
| 8 | Donkey |  |
| 9 | Mule |  |
| 10 | Horse |  |
| 11 | Hen |  |
| 12 | Chickens |  |
| 14 | Bull |  |
| 15 | Others |  |

1. **Inputs used for Onion production**

| Inputs | Amount (quantity) in Qt |
| --- | --- |
| Seed |  |
| DAP |  |
| Urea |  |

1. Frequency of extension personnel contact with the household head during the production season of Onion:
2. More than four times
3. Four times
4. Three times
5. Twice
6. Once
7. Not at all
8. Other (specify
9. Slope of farm plot
10. Steep
11. Plain
12. Frequency of watering per 15 days:-
13. Every day
14. 1X
15. 2X
16. 3X
17. Other, specify
18. Did you ever receive any training related to Onion production?
19. Yes
20. No
21. Did you ever receive any training related to Onion product marketing?
22. Yes
23. No
24. Amount of Onion produced _____________ Qt
25. Total plot size use for onion production Timad (1ha= 4 timad)
26. Labor and oxen used for different farming practices of Onion production by the household head

| Activities | Pair oxen | | Participants | | | | | | Number of hired labor | |
| --- | --- | --- | --- | --- | --- | --- | --- | --- | --- | --- |
|  | Number | Hours/day | Children | | Men | | Women | |  |  |
|  |  |  | Number | Hours/day | Number | Hours/day | Number | Hours/day | Number | Hours/day |
| First Plowing |  |  |  |  |  |  |  |  |  |  |
| Second plowing |  |  |  |  |  |  |  |  |  |  |
| Third Plowing |  |  |  |  |  |  |  |  |  |  |
| Fourth Plowing |  |  |  |  |  |  |  |  |  |  |
| Fifth Plowing |  |  |  |  |  |  |  |  |  |  |
| Planting/sowing |  |  |  |  |  |  |  |  |  |  |
| First Ridging |  |  |  |  |  |  |  |  |  |  |
| Second Ridging |  |  |  |  |  |  |  |  |  |  |
| Third Ridging |  |  |  |  |  |  |  |  |  |  |
| Watering |  |  |  |  |  |  |  |  |  |  |
| Harvesting |  |  |  |  |  |  |  |  |  |  |
| Others |  |  |  |  |  |  |  |  |  |  |

**Key**

1. Number (1, 2…)

2. Time (Hrs)

3. Age: - 01 if <10; 02 if 10-13; 03 if 14-16; 04 if 17-50; 05 if >50

4=Sex; F if female & M if male

**ጎንደር ዩኒቨርስቲ**

**የግብርናና ገጠር ትራንስፎርሜሽን ኮሌጅ**

**ግብርና ምጣኔ ሃብት ት/ክፍል**

ውድ ተሳታፊያችን፡-ይህ መጠይቅ የተዘጋጀው **“**በአማራ ክልል ሰሜን ጎንደር ዞን የመስኖ የቀይ ሽንኩርት ቴክኒካል ውጤታማነት ትንተና**”** በሚል ለማካሄድ ነው**፡፡**ለእያንዳንዱ ጥያቄ የምትሰጠው(ጪው) መልስ ለምርምር ብቻ የሚያገለግልና ለሦሥተኛ ወገን ይፋ አይሆንም (ተላልፎ አይሰጥም)፡፡ ለጥያቄዎች መልስ ለመስጠት ስለተባበሩን እናመሰግናለን፡፡

1. ስም _________________
2. ዕድሜ _____________ (ዓመት)
3. የቤተሰቡ ኃላፊ መደበኛ የትምህርት ዓመታት የክፍል ደረጃ? ______
4. የቀይ ሽንኩርትን ለምን ያህል አመታት አምርተዋል? _____
5. የቤተሰብ ሁኔታ(መረጃ)

| ፆታ | ዕድሜ ሥርጭት | | | | | ምርመራ |
| --- | --- | --- | --- | --- | --- | --- |
|  | <10 | 10-13 | 14-16 | 17-50 | >50 |  |
| ወንድ |  |  |  |  |  |  |
| ሴት |  |  |  |  |  |  |

1. የእንሰሳት አይነትና ብዛት

| ተ/ቁ | የእንሰሳት አይነት | ጠቅላላ ብዛት |
| --- | --- | --- |
| 1 | ላም |  |
| 2 | በሬ |  |
| 3 | ጊደር |  |
| 4 | ጥጃ |  |
| 5 | በግ |  |
| 6 | ወይፈን |  |
| 7 | ፍየል |  |
| 8 | አህያ |  |
| 9 | በቅሎ |  |
| 10 | ፈረስ |  |
| 11 | ሴት ዶሮ |  |
| 12 | አውራ ዶሮ |  |
| 14 | ኮርማ |  |
| 15 | ሌሎች |  |

1. ለቀይ ሽንኩርት የዋለ የግብዓት አይነት እና መጠን

| የግብዓት አይነት | የግብአት መጠን (ኩ/ል) |
| --- | --- |
| ዘር |  |
| ዳፕ |  |
| ዩሪያ |  |

1. የቀይ ሽንኩርት የምርት ዘመን ከግብርና ባለሙያ ጋር የነበርዎት ግንኙነት
2. ከ4 ጊዜ በላይ
3. 4 ጊዜ
4. 3 ጊዜ
5. 2 ጊዜ
6. አንድ ጊዜ
7. የለኝም
8. ሌላ ካለ ይጠቀስ
9. የማሳ ግድለት
10. ተዳፋት
11. ሜዳ
12. ቀይ ሽንኩርት በመስኖ ስታመርቱ በ15 ቀን ስንት ጊዜ ውሃ ታጠጣለህ/ሽ፡-
13. በየቀኑ
14. 1 ጊዜ
15. 2 ጊዜ
16. 3 ጊዜ
17. ሌላ ካለ ይጠቀስ____
18. በቀይ ሽንኩርት አመራረት ዙሪያ ስልጠና አግኝተሃል/ሻል?
19. ዎ 0) አይደለም
20. በቀይ ሽንኩርት ግብይት ዙሪያ ስልጠና አግኝተሃል/ሻል?
21. አዎ 0) አይደለም
22. የቀይ ሽንኩርት ምርት መጠን በኩ/ል ምን ያህል ነው? _____________ ኩ/ል
23. ለቀይ ሽንኩርት ምርት የዋለ አጠቃላይ የማሳ ስፋት _____ በጥማድ (1 ሄ/ር=4ጥማድ)
24. ለቀይ ሽንኩርት ምርት ከመሬት ዝግጅት እስከ ምርት ስበሰባ የዋለ የሠውና የበሬ ጉልበት መጠን

| ዝርዝር ተግባር | ጥንድ በሬ | | ተሳታፊዎች | | | | | | | | | የተቀጠሩ ሠራተኞች ብዛት (ወንድ) | |
| --- | --- | --- | --- | --- | --- | --- | --- | --- | --- | --- | --- | --- | --- |
|  | ቁጥር | ሠዓት/ቀን | ህፃናት | | | | | ወንድ | | ሴት | |  |  |
|  |  |  | ወንድ | | ሴት | | |  |  |  |  |  |  |
|  |  |  | ቁጥር | ሠዓት/ቀን | | ቁጥርr | ሠዓት/ቀን | ቁጥር | ሠዓት/ቀን | ቁጥር | ሠዓት/ቀን | ቁጥር | ሠዓት/ቀን |
| የመጀመሪያ እርሻ |  |  |  |  | |  |  |  |  |  |  |  |  |
| 2ኛ እርሻ |  |  |  |  | |  |  |  |  |  |  |  |  |
| 3ኛ እርሻ |  |  |  |  | |  |  |  |  |  |  |  |  |
| 4ኛ እርሻ |  |  |  |  | |  |  |  |  |  |  |  |  |
| 5ኛ እርሻ |  |  |  |  | |  |  |  |  |  |  |  |  |
| ተከላ/መዝራት |  |  |  |  | |  |  |  |  |  |  |  |  |
| 1ኛ ኩትኮታና አረም |  |  |  |  | |  |  |  |  |  |  |  |  |
| 2ኛ ኩትኮታና አረም |  |  |  |  | |  |  |  |  |  |  |  |  |
| 3ኛ ኩትኮታና አረም |  |  |  |  | |  |  |  |  |  |  |  |  |
| ውሃ ማጠጣት |  |  |  |  | |  |  |  |  |  |  |  |  |
| መሰብሰብ |  |  |  |  | |  |  |  |  |  |  |  |  |
| ሌላ ካለ ይጠቀስ |  |  |  |  | |  |  |  |  |  |  |  |  |

**መፍቻ**

1. ቁጥር(1, 2…)

2. ጊዜ (ሠዓት)

3. ዕድሜ: - 01 ከ <10; 02 በ 10-13; 03 በ 14-16; 04 በ 17-50; 05 ከ >50

4=ፆታ; ሴ =ሴት & ወ=ወንድ
