# Supplementary material for: Analysis of technical efficiency of irrigated onion (Allium cepa L.) production in North Gondar Zone of amhara regional state, Ethiopia
Source: PLoS One. 2022 Oct 13;17(10):e0275177. doi: 10.1371/journal.pone.0275177 (PMC9562163; doi:10.1371/journal.pone.0275177)
Supplement: S1 Appendix — (DOCX) [file pone.0275177.s003.docx]

Appendixes

Appendix Table 1: VIF of Explanatory Variables of Stochastic Frontier Production Function Model

| **Variables** | $\mathbf{VIF}$ | $\frac{\mathbf{1}}{\mathbf{VIF}}$ |
| --- | --- | --- |
| LnDAP | 1.76 | 0.57 |
| LnODE | 1.67 | 0.60 |
| Lnplotsize | 1.56 | 0.64 |
| LnUrea | 1.50 | 0.67 |
| LnMDE | 1.43 | 0.70 |
| LnSEED | 1.28 | 0.78 |
| Mean VIF | 1.53 |  |

Source: Computed from Field Survey Data, 2015/16

Appendix Table 2: VIF for continuous variables used to technical inefficiency model (n=205)

| **Variables** | $\mathbf{VIF}$ | $\mathbf{1}/\mathbf{VIF}$ |
| --- | --- | --- |
| Famsze | 1.37 | 0.73 |
| Extfrqnt | 1.33 | 0.75 |
| Age | 1.33 | 0.75 |
| Educ | 1.24 | 0.81 |
| Waterfrnq | 1.22 | 0.82 |
| TLU | 1.12 | 0.89 |
| Exp | 1.09 | 0.92 |
| Mean VIF | 1.24 |  |

Source: Computed from Field Survey Data, 2015/16

Appendix Table 3: Contingency coefficients for hypothesized discrete explanatory variables (n=205)

|  | Trngp | Trngm | Slope |
| --- | --- | --- | --- |
| Trngp | 1 | 0.459 | 0.065 |
| Trngm |  | 1 | 0.096 |
| Slope |  |  | 1 |

Source: Computed from Field Survey Data, 2015/19

Appendix Table 4: MLE of Parameters of trans-log stochastic production Frontier Function for onion Producers

| **Variables** | **Coefficient** | **standard-error** | **t-ratio** |
| --- | --- | --- | --- |
| Constant | -10.63 | 6.98 | -1.52 |
| LnODE | -0.29 | 1.31 | -0.22 |
| LnMDE | 1.05 | 0.96 | 1.10 |
| LnSD | 0.97 | 0.91 | 1.06 |
| LnDAP | 1.83 | 1.07 | 1.70 |
| LnUrea | 0.42 | 0.98 | 0.43 |
| LnPlot | 1.57 | 1.60 | 0.98 |
| $\mathrm{Ln}\mathrm{ODE}^{2}$ | -0.29 | 0.12 | -2.31 |
| $\mathrm{Ln}\mathrm{MDE}^{2}$ | -0.01 | 0.07 | -0.18 |
| $\mathrm{Ln}\mathrm{SD}^{2}$ | -0.02 | 0.04 | -0.48 |
| $\mathrm{Ln}\mathrm{DAP}^{2}$ | 0.02 | 0.05 | 0.35 |
| $\mathrm{Ln}\mathrm{Urea}^{2}$ | -0.04 | 0.04 | -0.96 |
| $\mathrm{Ln}\mathrm{PLot}^{2}$ | 0.16 | 0.13 | 1.27 |
| LnODE_*_LnMDE | -0.01 | 0.13 | -0.11 |
| LnODE*LnSD | -0.08 | 0.12 | -0.63 |
| LnODE*LnDAP | 0.33 | 0.12 | 2.78 |
| LnODE*LnUrea | -0.16 | 0.09 | -1.82 |
| LnODE*LnPlot | 0.31 | 0.17 | 1.88 |
| LnMDE*LnSD | 0.16 | 0.08 | 1.97 |
| LnMDE*LnDAP | 0.04 | 0.10 | 0.35 |
| LnMDE*LnUrea | 0.05 | 0.09 | 0.62 |
| LnMDE*LnPlot | -0.30 | 0.13 | -2.36 |
| LnSD*LnDAP | 0.08 | 0.07 | 1.13 |
| LnSD*LnUrea | -0.05 | 0.07 | -0.68 |
| LnSD*LnPlot | -0.18 | 0.10 | -1.79 |
| LnDAP*LnUrea | 0.03 | 0.07 | 0.36 |
| LnDAP*LnPlot | -0.48 | 0.15 | -3.18 |
| lnUrea*LnPlot | 0.03 | 0.11 | 0.29 |
| Efficiency factors |  |  |  |
| Constant | 0.17 | 1.80 | 0.10 |
| Age | 0.18 | 0.50 | 0.37 |
| Educ | 0.16 | 0.09 | 1.94 |
| FAM | 0.10 | 0.05 | 2.03 |
| TLU | -0.05 | 0.03 | -1.77 |
| EXP | -0.09 | 0.04 | -2.29 |
| EXTF | 0.14 | 0.26 | 0.52 |
| SLOPE | 0.04 | 0.29 | 0.15 |
| TRGP | -0.57 | 0.30 | -1.87 |
| TRGM | 0.82 | 0.36 | 2.25 |
| WTRF | -0.97 | 0.29 | -3.32 |
| Variance parameters |  |  |  |
| Sigma-squared$(\sigma^{2})$ | 0.65 | 0.16 | 3.98 |
| Gamma$\left( \gamma\right)$ | 0.81 | 0.09 | 9.42 |
| log-likelihood function | -185.31 |  |  |
| LR | 63.59 |  |  |
| Total sample size | 205 |  |  |

Source: Computed from Field Survey Data, 2015/16
