# Supplementary material for: Analysis of technical efficiency of irrigated onion (Allium cepa L.) production in North Gondar Zone of amhara regional state, Ethiopia
Source: PLoS One. 2022 Oct 13;17(10):e0275177. doi: 10.1371/journal.pone.0275177 (PMC9562163; doi:10.1371/journal.pone.0275177)
Supplement: Attachment — Submitted filename: PONE-D-22-07294_reviewer.pdf [file pone.0275177.s004.pdf]

PLOS ONE

Analysis of Technical Efficiency of Irrigated Onion (Allium cepa L.) Production in North Gondar Zone of Amhara Regional State, Ethiopia

--Manuscript Draft--

**Manuscript Number:**	PONE-D-22-07294
**Article Type:**	Research Article
**Full Title:**	Analysis of Technical Efficiency of Irrigated Onion (Allium cepa L.) Production in North Gondar Zone of Amhara Regional State, Ethiopia
**Short Title:**	Analysis of Technical Efficiency of Irrigated Onion (Allium cepa L.) Production
**Corresponding Author:**	Tigabu Dagnew Koye, MSc in Agricultural Economics<br>University of Gondar<br>Gondar, Amhara region ETHIOPIA
**Keywords:**	technical efficiency, stochastic frontier, Cobb Douglas, onion, North Gondar Zone
**Abstract:**	Onion plays an important role in contributing to a household's food security and income. But, limited improved seeds, low fertilizers application, low marketing infrastructure, weak institutional support, and market access have contributed to onion's low productivity. Therefore, this study tries to fill the gap by investigating efficiency variations and identifying the factors that influence the efficiency levels of onion producers in the North Gondar Zone. Primary and secondary data sources were used. A semi-structured interview schedule was employed to collect primary data from 205 onion producers selected through simple random sampling proportional to sample size from Gondar Zuria, Takusa, and Dembia Districts. To examine the farm-level technical efficiency of onion production; descriptive statistics, the single-stage stochastic frontier model, and Cobb Douglas production function were applied. The maximum likelihood estimates of the stochastic frontier analysis indicated that the mean technical efficiency of irrigated onion was 53%. The implication of the mean production efficiency of irrigated onion could be increased through improving farming practices with the existing technology. The maximum likelihood estimates of the stochastic frontier model showed that plot size, DAP, and oxen have a significant effect on onion output; and education, livestock holding, experience, and watering frequency have a positive and significant effect on technical efficiency, whereas family size and training on marketing have a negative and significant effect on technical efficiency; Therefore, governmental and/or private institutions should work to improve the production of the onion by providing the necessary institutional support to the smallholder farmers in the study areas.
**Order of Authors:**	Tigabu Dagnew Koye, MSc in Agricultural Economics
	Abebe Dagnew Koye, PhD candidate in Agricultural Economics
	Zework Aklilu Amsalu, PhD Candidate in Economics
**Additional Information:**	
**Question**	**Response**
**Financial Disclosure**<br><br>Enter a financial disclosure statement that describes the sources of funding for the work included in this submission. Review the submission guidelines for detailed requirements. View published research articles from *PLOS ONE* for specific examples.<br><br>This statement is required for submission	This study has been funded by the University of Gondar

Powered by Editorial Manager® and ProduXion Manager® from Aries Systems Corporation

and **will appear in the published article** if the submission is accepted. Please make sure it is accurate.<br><br>**Unfunded studies**<br>Enter: *The author(s) received no specific funding for this work.*<br><br>**Funded studies**<br>Enter a statement with the following details:<br>• Initials of the authors who received each award<br>• Grant numbers awarded to each author<br>• The full name of each funder<br>• URL of each funder website<br>• Did the sponsors or funders play any role in the study design, data collection and analysis, decision to publish, or preparation of the manuscript?<br>• **NO** - Include this sentence at the end of your statement: *The funders had no role in study design, data collection and analysis, decision to publish, or preparation of the manuscript.*<br>• **YES** - Specify the role(s) played.<br><br>* typeset	
**Competing Interests**<br><br>Use the instructions below to enter a competing interest statement for this submission. On behalf of all authors, disclose any competing interests that could be perceived to bias this work—acknowledging all financial support and any other relevant financial or non-financial competing interests.<br><br>This statement is required for submission and **will appear in the published article** if the submission is accepted. Please make sure it is accurate and that any funding sources listed in your Funding Information later in the submission form are also declared in your Financial Disclosure statement.<br><br>View published research articles from *PLOS ONE* for specific examples.	The authors have declared that no competing interests exist.

**NO authors have competing interests**<br><br>Enter: *The authors have declared that no competing interests exist.*<br><br>**Authors with competing interests**<br><br>Enter competing interest details beginning with this statement:<br><br>*I have read the journal's policy and the authors of this manuscript have the following competing interests: [insert competing interests here]*<br><br>* typeset	
**Ethics Statement**<br><br>Enter an ethics statement for this submission. This statement is required if the study involved:<br><br>• Human participants<br>• Human specimens or tissue<br>• Vertebrate animals or cephalopods<br>• Vertebrate embryos or tissues<br>• Field research<br><br>Write "N/A" if the submission does not require an ethics statement.<br><br>General guidance is provided below. Consult the submission guidelines for detailed instructions. **Make sure that all information entered here is included in the Methods section of the manuscript.**	N/A

**Format for specific study types**<br><br>**Human Subject Research (involving human participants and/or tissue)**<br>• Give the name of the institutional review board or ethics committee that approved the study<br>• Include the approval number and/or a statement indicating approval of this research<br>• Indicate the form of consent obtained (written/oral) or the reason that consent was not obtained (e.g. the data were analyzed anonymously)<br><br>**Animal Research (involving vertebrate animals, embryos or tissues)**<br>• Provide the name of the Institutional Animal Care and Use Committee (IACUC) or other relevant ethics board that reviewed the study protocol, and indicate whether they approved this research or granted a formal waiver of ethical approval<br>• Include an approval number if one was obtained<br>• If the study involved *non-human primates*, add *additional details* about animal welfare and steps taken to ameliorate suffering<br>• If anesthesia, euthanasia, or any kind of animal sacrifice is part of the study, include briefly which substances and/or methods were applied<br><br>**Field Research**<br><br>Include the following details if this study involves the collection of plant, animal, or other materials from a natural setting:<br>• Field permit number<br>• Name of the institution or relevant body that granted permission	
**Data Availability**<br><br>Authors are required to make all data underlying the findings described fully available, without restriction, and from the time of publication. PLOS allows rare exceptions to address legal and ethical concerns. See the PLOS Data Policy and FAQ for detailed information.	Yes - all data are fully available without restriction

A Data Availability Statement describing where the data can be found is required at submission. Your answers to this question constitute the Data Availability Statement and **will be published in the article**, if accepted.<br><br>**Important:** Stating 'data available on request from the author' is not sufficient. If your data are only available upon request, select 'No' for the first question and explain your exceptional situation in the text box.<br><br>Do the authors confirm that all data underlying the findings described in their manuscript are fully available without restriction?	
**Describe where the data may be found in full sentences. If you are copying our sample text, replace any instances of XXX with the appropriate details.**<br><br>• If the data are **held or will be held in a public repository**, include URLs, accession numbers or DOIs. If this information will only be available after acceptance, indicate this by ticking the box below. For example: *All XXX files are available from the XXX database (accession number(s) XXX, XXX.).*<br>• If the data are all contained **within the manuscript and/or Supporting Information files**, enter the following: *All relevant data are within the manuscript and its Supporting Information files.*<br>• If neither of these applies but you are able to provide **details of access elsewhere**, with or without limitations, please do so. For example:<br><br>*Data cannot be shared publicly because of [XXX]. Data are available from the XXX Institutional Data Access / Ethics Committee (contact via XXX) for researchers who meet the criteria for access to confidential data.*<br><br>*The data underlying the results presented in the study are available from (include the name of the third party*	This text is appropriate if the data are owned by a third party and authors do not have permission to share the data.

and contact information or URL).<br>• This text is appropriate if the data are owned by a third party and authors do not have permission to share the data.<br><br>* typeset	
Additional data availability information:	Tick here if the URLs/accession numbers/DOIs will be available only after acceptance of the manuscript for publication so that we can ensure their inclusion before publication.; Tick here if your circumstances are not covered by the questions above and you need the journal's help to make your data available.

Manuscript Click here to access/download;Manuscript;Manuscript .docx

Analysis of Technical Efficiency of Irrigated Onion (*Allium cepa* L.) Production in North Gondar Zone of Amhara Regional State, Ethiopia

Tigabu Dagnew Koye[1], Abebe Dagnew Koye[1] and Zework Aklilu Amsalu[1]

[1]Department of Agricultural Economics, College of Agriculture and Environmental Science, University of Gondar, Gondar, Ethiopia, Email: tigabu567@gmail.com, adagnew268@gmail.com, and zeworkamsalu@gmail.com, respectively

ABSTRACT

Onion plays an important role in contributing to a household's food security and income. But, limited improved seeds, low fertilizers application, low marketing infrastructure, weak institutional support, and market access have contributed to onion's low productivity. Therefore, this study tries to fill the gap by investigating efficiency variations and identifying the factors that influence the efficiency levels of onion producers in the North Gondar Zone. Primary and secondary data sources were used. A semi-structured interview schedule was employed to collect primary data from 205 onion producers selected through simple random sampling proportional to sample size from Gondar Zuria, Takusa, and Dembia Districts. To examine the farm-level technical efficiency of onion production; descriptive statistics, the single-stage stochastic frontier model, and Cobb Douglas production function were applied. The maximum likelihood estimates of the stochastic frontier analysis indicated that the mean technical efficiency of irrigated onion was 53%. The implication of the mean production efficiency of irrigated onion could be increased through improving farming practices with the existing technology. The maximum likelihood estimates of the stochastic frontier model showed that plot size, DAP, and oxen have a significant effect on onion output; and education, livestock holding, experience, and watering frequency have a positive and significant effect on technical efficiency, whereas family size and training on marketing have a negative and significant effect on technical efficiency; Therefore, governmental and/or private institutions should work to improve the production of the onion by providing the necessary institutional support to the smallholder farmers in the study areas.

This problem is a regional issue.

,Ethiopia

show the full name of DAP

Keywords: technical efficiency, stochastic frontier, Cobb Douglas, onion, North Gondar Zone

1. INTRODUCTION

Vegetables are non woody plants and plant components that generally may be consumed raw or with smallest change of state (Raman, 2011). Vegetables may be bulb; fruity; inflorescent; leafy; root and stalk. Vegetables may be fully grown in an exceedingly wide selection of climates with extreme heat, cold, excessive rainfall/, or drought (Gambo, 2008). Vegetables form a vital element of our daily food. They assist in protecting the body against diseases. Most vegetables are low in fat and calories and many of them are good sources of fiber. The high level of fiber in vegetables keeps the digestive system healthy and prevents constipation. The nutritional and health worth of vegetables is additionally well recognized in Ethiopia as a result of vegetables play key roles in human health by means of providing antioxidants like vitamin A, C, and E that are vital in neutralizing free radicals (oxidants) better-known to cause cancer, cataracts, cardiovascular disease, high blood pressure, stroke, and polygenic disease (Demissie *et al*., 2009; Tabor and Yesuf, 2012). Onion (Allium Cepa) is a vital vegetable crop that's a complimentary product to tomatoes and is of major commercial importance throughout the planet. According to USDA (2009), approximately 200 million tons of dry bulbs onions are produced in 2009 in the world of which 74.9 million tons were produced in America alone leaving Asia, Europe, and Africa with 125.1 million tones.

Delete these sentences

the recent data need to be shown

Horticultural crops normally and vegetables, specifically, /play a vital role in contributing to home food security and financial gain. The vegetables being money crops with nutritional value generate financial gain for the poor households. Higher profits are often achieved by increasing the production of a selected vegetable throughout the year when an efficient economical irrigation system is employed Agumas *et al*. (2014). Horticultural production is sometimes money-spinning as compared to staple crops. The production of fruits and vegetables encompasses a comparative advantage significantly underneath conditions wherever cultivable land is scarce, labor is ample and markets are accessible (Getahun, 2014). Vegetable crops of economic importance that are mostly created in Federal Democratic Republic of Ethiopia include pepper, kale (Ethiopian cabbage), onion, tomato, chilies, carrot, garlic, and cabbages (Ethiopian Investment Agency, 2012). The production of horticultural products offers opportunities for poverty alleviation/ because it is usually more labor-intensive than the production of staple food crops. Hence, the generation of additional employment opportunities in rural areas where labor is abundant is created achievable.

Vegetable products provide nutritional, economic, employment, and social benefits. Vegetable production and consumption are increasing in Ethiopia because of increasing export to Djibouti, Somalia, South Sudan, Sudan, the Middle East, and European markets and urbanization (Tabor and Yesuf, 2012). In these countries there is a sustained demand for products such as chilies, onions, and cabbages, resulting in an export increase from 25,300 tons in 2002/03 to 63,140 tons in 2009/10 (EHDA, 2011).

In Ethiopia, it's associate economically important place among different vegetable crops due to the ease of production, high profitability per unit area and increase in small-scale irrigation schemes, the area coverage of the onion production is increasing over time. According to CSA (2015), the productivity of onion was 10.05 and 10.13 t/ha in 2013 and 2014/2015, respectively. Area coverage of onion in the country increased to 33, 063.37 ha, and 327,475.25 tons were obtained in 2016/2017 which correspond to 9.74 tons/ha (CSA, 2015). Despite its importance in the human diet, economic profit and its increasing area coverage, the productivity of onion in the country is much lower than of other African countries with an average value of 10.1 ton/ha (CSA, 2015).

In the Amhara region irrigated agriculture is increasing in recent years. Currently, over 6,200 small-scale irrigation schemes of which 95% are traditional exist within the region (Melisew, 2012). Apparently, the irrigation schemes are owned by quite 330,000 households (or more than 1.9 million people) with an average irrigated land holding of 0.2 ha (Melisew, 2012). Since farmers used improper agronomic practices, limited improved seeds, and fertilizers which have an excellent contribution to lowering the productivity of onion (Yemane *et al.*, 2016). Moreover, Addisu (2017) reported that promoting infrastructure, institutional support, and market access contributed to onion's low productivity. It is difficult to extend onion production by increasing the area of land under cultivation due to the limitation of land. But, there is a chance to extend the production of the onion by improving the prevailing production technology. Farmers could also be comparatively inefficient due to less experience, illiteracy, etc. If farmers are found to be technically inefficient, production will be redoubled to an oversized extent using the prevailing level of agricultural inputs, agricultural extension services, and obtainable technology. Theoretically, productivity will be redoubled through disseminating new technology and/or improving the production efficiency of the smallholder farmers. The primary possibility is costly since there is a shortage of agricultural technology generally and onion above all. Hence, improving productivity through enhancing production efficiency using the prevailing resources is that the best for smallholder onion farmers.

In the past, so far, the author's knowledge is concerned; in the study areas, there have been no similar studies on the technical efficiency of onion producers. A number of the studies conducted are Abate *et al*. (2019) on the Technical efficiency of smallholder farmers in red pepper production in North Gondar Zone; Wassihun *et al.* (2019) on Analysis of technical efficiency of potato (*Solanum tuberosum* L.) production in Chilga District, Amhara National Regional State, Ethiopia; Asfaw (2021) on Analysis of technical efficiency of smallholder tomato producers in Asaita district, Afar National Regional State, Ethiopia; Abdulkadir (2015) on an evaluation of the efficiency of onion producing farmers in irrigated agriculture: Empirical evidence from Kobo district, Amhara region, Ethiopia; Aman *et al*. (2019) on Vegetable Production Efficiency of Smallholders' Farmer in West Shewa Zone of Oromia National Regional State, Ethiopia. Hence, this analysis was conducted to fill this gap to estimate technical efficiency among small-scale irrigated onion-producing farmers and to identify determinant factors on the technical efficiency of onion producers in the North Gondar Zone.

2. RESEARCH METHODOLOGY

Materials and methods

2.1.Description of the Study Area

It is better to present a map of study area

The study was conducted in North Gondar Zone, Amhara regional state, Ethiopia. It is located in the northwestern part of Ethiopia between 11 and 13 north latitudes, and 35 and 35 east longitudes, and it is 783 km far from Addis Ababa. The zonal capital city is Gondar, and geographically Gondar is located at 12°35′ 60.00″N latitude and 37°28′ 0.01″E longitudes with an average elevation of 2133 meters above sea level. The zone is dominated by the agricultural sector, which employs about 90% of the working force. The boundaries are adjoined with the Tigray region in North, Awi zone and West Gojam zone in South, Waghimra zone in East, South Gondar zone in Southeast, and Sudan in West. The total area of the zone is 50,970 Square Km Abate *et al*. (2019). The study was conducted in Gondar Zuria, Dembia, and Takusa Districts. The crop production systems are characterized by rain-fed and irrigation. According to the zonal department of agriculture, farmers used irrigation mainly for vegetable production such as onion, tomato, cabbage, pepper, potato, etc and very often cereal such as maize and the like. According to the zonal department of agriculture, onion takes the lion's share in terms of irrigated land allocated and volume of production.

2.2. Sampling Technique and Sample Size

To select respondent producers, a multi-stage sampling technique was used. In the first stage, out of the total woredas of North Gondar Zone, three woredas namely Takusa, Dembia, and Gondar Zuria were selected purposively based on their production potentials (better water and irrigable farm access, a better volume of production, and more number of producers). In the second stage, Chemera, Chanikie, and Mekonta from Takusa woreda; Abrjeha and Sufankara from Dembia woreda; Sendeba and Ambober from Gondar Zuria woreda were selected randomly based on proportion to the total number in woredas. Finally, 205 irrigated onion producers were selected randomly proportional to the number of farmers.

2.3. Data Type, Sources, and Method of Data Collection

Both primary and secondary data were used for the study. Primary data was collected through personal and face-to-face interviews using a semi-structured and pre-tested interview schedule that was filled up by recruited and trained enumerators under close supervision. Secondary data was obtained from various sources such as reports of the bureau of agriculture at different levels, NGOs, CSA, District administrative office, previous research findings, internet, and other published and unpublished materials, which were relevant to the study.

2.4. Methods of Data Analysis and model specifications

The data were analyzed through descriptive statistics (such as percentage, frequency, minimum, maximum, mean, and standard deviation). For econometrics analysis (namely stochastic frontier), a single-stage stochastic frontier model was used to estimate the level of efficiency and analysis the determinants of technical efficiency of small-scale irrigated onion producers.

The variation of actual output from the frontier due to inefficiency and random shocks can be captured through the stochastic frontier approach. The existence of inefficiency in crop production comes from the inefficient use of scarce resources. There exist two main competing methods for analyzing technical efficiency and its principal determinants: the parametric frontier (stochastic frontier approach) and the non-parametric frontier (data envelopment analysis) Shumet (2011). The non-parametric frontier suffers from the criticism that it takes no account of the possible influence of random shocks like measurement errors and other noises in the data (Coelli, 1995).

The reason to settle on a stochastic frontier model was, it is appropriate for analyzing farm-level data where measurement errors are substantial and therefore the weather (natural hazards, surprising climatic conditions, pest, and disease) is probably going to possess a big impact (Dessale, 2019, Coelli and Battese, 1995) and it is also referred to as the econometric frontier approach, specifies a functional form for the cost, profit, or production relationship among inputs, outputs, and environmental factors, and it allows for random errors Mekonnen *et al.* (2015). The Stochastic Frontier Approach (SFA) was developed independently by Aigner *et al*. (1977) and Meeusen and Van der Broeck (1977). SFA approach can be extended to measure inefficiencies in individual production units based on some distributional assumptions for the technical and economic inefficiency scores Asfaw DM (2021), Alemu *et al* (2018).

Several functional forms have been developed to measure the physical relationship between input and output, and the most common functional forms are Cobb-Douglas and transcendental logarithmic (translog) function as Wassihun *et al*. (2019). The Cobb-Douglas is simpler but less flexible, the form is very parsimonious concerning degrees of freedom (Leavy *et al*., 1999) and it meets the requirement of being self-dual which allows the examination of allocative and economic efficiency.

However, one of the drawbacks of Cobb-Douglas is that it's less versatile because it imposes a severe previous restriction on the farm's technology by limiting the production elasticity to be constant and the elasticity of input substitution to unity (Wilson and Hadley, 1998).

The trans-log production function on the other hand is a more flexible functional form than the Cobb-Douglas, which takes into account the interactions between variables and allows for non-linearity in the parameters. However, the trans-log suffers some drawbacks. First, it does not yield coefficients of a plausible sign and magnitude due to the degrees of freedom, and secondly, when estimating trans-log production function, multicollinearity among explanatory variables is usually present (Leavy *et al.,* 1999). From the above literature review one can understand that unless we use a test of hypothesis to choose either of them, no one can say one better than the other. In this study test of the hypothesis was employed to select either of them. Likelihood Ratio Test (LR Test) may be conducted to test the model specification of stochastic production frontier. The null hypothesis of the LR Test is that all the interactions and second-order terms regarding trans-log specification equal zero Morais *et al*. (2021).

The Cobb-Douglas has been wide utilized in several empirical studies, significantly those associated with developing countries for farm efficiency analysis (Bravo-Ureta and Pinheiro, 1997). The function is formulated as:-

$$Y_i = F(X_i\beta)\exp(v_i - u_i) \quad i = 1,2,3,\ldots,205 \tag{1}$$

Where: Yi = Onion output, i = the ith farmer in the sample, Xi = a vector of inputs used by the ith farmer, β = a vector of unknown parameters, Vi = a random variable which is assumed to be normally and independently distributed, and Ui = farm-specific technical inefficiency in production and nonnegative random variable. The Cobb–Douglas form of stochastic frontier production is stated as:

$$\ln Y = \beta_0 + \sum_{j=1}^{6} \beta_j \ln X_{ij} + v_i - u_i \tag{2}$$

Where: ln = natural logarithm, X_{ij} = is the quantity of input j used in the production process including oxen (ODE), labor (MDE), plot size (ha), DAP (kg), Urea (kg), and seed (kg). After estimating the technical inefficiency (u_i) from Eq (2), the technical inefficiency model was specified as:

$$\mu_i = \delta_0 + \delta_{1i}(Age_i) + \delta_{2i}(Educ_i) + \delta_{3i}(Famsze_i) + \delta_{4i}(TLU_i) + \delta_{5i}(Expr_i) + \delta_{6i}(Frqcnt_i) + \delta_{7i}(Slope_i) + \delta_{8i}(Trngprdn_i) + \delta_{9i}(Trngmkt_i) + \delta_{10i}(wateringfrnq_i) \tag{3}$$

Where: The subscript i, indicates the ith household in the sample; $\delta_0, \delta_{1i}, \ldots, \delta_{10i}$ are parameters to be estimated.

The farm-specific technical efficiency is defined in terms of observed output (Y_i) to the corresponding frontier output (Y_i^*) using the available technology was specified as:

$$TE_i = \frac{Y_i}{Y_i^*} = \frac{E(Y_i/U_i, X_i)}{E(Y_i/U_i = 0, X_i)} = E[\exp(-U_i)/\varepsilon_i] \tag{4}$$

TE takes the value on the interval (0, 1), where 1 indicates a fully efficient farm.

The maximum likelihood estimates for the parameters of the stochastic frontier are gained by applying the Frontier 4.1 (Coelli, 1996) computer program, in which the variance parameters are expressed in terms of:

$$\sigma^2 = \delta_v^2 + \delta_\mu^2 \text{ and} \tag{5}$$

$$\gamma = \sigma_\mu^2 \big/ (\sigma_v^2 + \sigma_\mu^2) \tag{6}$$

Where: σ^2 it is the total variance of the model and the term γ represents the ratio of the variance of inefficiency's error term to the total variance of the two error terms defined above. The value of variance parameter γ ranges between 0 and 1.

2.5.Variable Definitions and Expected Signs

In technical efficiency analysis, there are input-output variables and technical efficiency versus demographic and socioeconomic variables.

Dependent variable: was the total physical output of onion in kilogram (local measurement was used and then converted into the scientific measurement unit) obtained from the total area of irrigated onion plot in the production year.

Independent variables: are inputs used in onion production and Producer's socio-economic variables those influence technical efficiency were defined as follows:

Plot size: The total area of irrigated plot in ha allocated for onion production by the ith household. It is expected to determine the efficiency differential of farmers in the study area. It is important to evaluate whether relatively large farms are more efficient or not than small ones. As the farm size of a farmer increases, the manageability may decrease. The land is the main factor of production and thus positive coefficient is expected (Bekele, 2013).

Fertilizer: Total amount of fertilizer (DAP and urea) applied by the ith household on irrigated onion production measured in kg. The amount of fertilizer was expected to have a positive effect on yield, but when an overdose happens it can lead to low yield or total crop failure. Therefore, the application of chemical fertilizer will increase the level of production and the efficiency level of the farmer will be positive (Alemayehu, 2010).

Oxen power: the total number of oxen days used by the ith household from land preparation for onion seedling development and seedling transplanted. The amount of draught power used for different farming activities was taken by considering the number of oxen power used in oxen day. If the farmer has enough pairs of oxen with which to plow, he can accomplish his operations timely and with good quality. Hence, in this study, it was hypothesized that the more heads of oxen the farmers have the more productive they will be.

Labor: The overall labor force used for plowing, sowing, transplanting, weeding and cultivation (hoeing), fertilizer application, watering, and alternative pre-harvest management activities, which are all measured in terms of man-days. Family labor, exchange, and employed labor concerned within the production process, child labor, adult men and women, and aged labor were registered

singly and also the total weighed labor (Man Equivalent) in person-hours were measured in man-days employing a standard conversion factor. Given the fact that labor is the main input in production, a farmer that has been more labor within the household can carry out important crop husbandry practices timely (Abebe, 2009).

Seed: The total quantity of onion seed used by the ith household measured in kg. A very high seed density may result in low onion output due to high competition for nutrients, whereas a very low seed density may also lead to low onion output due to the under-utilization of the land. Hence, it was hypothesized that the quantity of seeds determines the seed rate which can have either positive or negative influence on yield.

Age: The age of producers captures variations within the quality of management. Well-versed farmers tend to reduce losses and have better managerial skills which might be used in their production method. Since farming experience increases with a rise within the number of years in farming, it was expected to have a positive effect on efficiency. As the age of the farmer increases, he/she will be able to use resources in such a way that it yields the maximum possible output.

Level of Education: Farmers who received additional education probably to possess have been exposed to information on the production of onion using technologies. Moreover, educated farmers are expected to possess higher capabilities in processing information and sorting out acceptable technologies to cut back inputs. Education of the farmer is predicted to cut back technical inefficiency.

Family size (adult equivalent)**:** This is a continuous variable representing the number of family members in the household. Family is an essential source of labor supply in the area. Household size can have a positive effect in raising the efficiency of the farmer in the production of onion. Since labor is the main input in the production as the farmer has a large family size, he/she was expected to manage onion plots on time (Fantu *et al.*, 2011). Thus, household size was hypothesized to have a positive influence on the production efficiency of the farmer.

Livestock holding: It refers to the number of livestock owned by the household and measured by the Tropical Livestock Unit (TLU). Livestock possession is perceived because the accumulation of wealth standing, use for draft power, manure, financial gain from the sale of butter, and sale of livestock in times of risk to shop improved agricultural technologies like seeds, pesticides, etc. Households having outsized livestock can have a better chance to earn more income from livestock. Therefore, the more livestock owned by the farm; the household will be the more possibility of

purchasing improved agricultural inputs and/or the more investment in the off-farm activity. Again the farmer also has the chance to get oxen for draught power (Getahun, 2014). Hence, those farmers owning more livestock were expected to be more technically efficient.

Experience: It is a continuous variable; measured in the number of years that the household head spends in farm activity. The number of years of experience is directly related to the farmers' knowledge of onion production. So, it was expected to affect the technical efficiency positively.

Watering frequency: Water availability is the main limiting factor of crop productivity over all of the rest due to its paramount importance for normal plant growth and development. Hence, due to its shallow root system and needs frequent irrigation water after a short interval, onion is susceptible to water stress as compared to other crops (Fitsum *et al*., 2016). Therefore, it is assumed farmers who avail irrigation water short frequently; was expected to affect technical efficiency positively.

Frequency of extension contact: Farmers who have better extension contact are expected to be more efficient than others. The more contact the farmer has with adding service, the more will be the information/knowledge she/he has and the better will be the use of agricultural inputs. Therefore, it is assumed that farmers who have frequent contact with Development Agents are more expected to demand agricultural inputs because of the increased awareness, and it was expected to affect the technical efficiency positively (Dawit, 2012).

Slope: The slope of the land may affect the level of production. Since steep plots are vulnerable to erosion damage and they are likely infertile compared to plain plots, slopes of the plot were found to be related negatively to technical efficiency (Alemayehu, 2010). It is a dummy variable that assumes a value of 1 if the slope of the plot is steep and 0 otherwise. It is hypothesized to determine the technical efficiency negatively.

Training on production and marketing: Training is an important tool in building the managerial capacity of the household head. Households' heads have gotten those who get training related to crop production and marketing or any related agricultural training is expected to be more efficient than those who did not receive training (Wondimu, 2013). It was expected to the technical efficiency positively.

3. RESULTS AND DISCUSSIONS

3.1. Descriptive analysis

kg/ha

The mean yield of onions produced by households was approximately 2,965kg per ha with a
what's the unit of this value?
standard deviation of 2,958. The mean plot size of the household was 0.36 hectares with a standard
what's the unit of this value?
deviation of 0.24. The onion producers are in the productive age since the average age was 44 years with a standard deviation of about 10. Family size on average was found to be 6 with a standard deviation of 2. Large family size together with small farmland and his/her management in the production system, it is difficult for the farmer to sustain his/her family. On average, extension workers made 3.4 visits to each in the production year, which facilitates new technology transfer. Training has a great contribution to irrigated onion production and marketing to boost the income of the farmers. To this end, among the household, about 65.37% and 86.34% reported that they were not trained in production and marketing, respectively. This indicates that training might not have an impact on the technical efficiency differentials among the household heads. In terms of watering frequency 17.07%, 48.78%, 33.17%, and 0.98% of the sample households irrigate (water) their onion plot two times, three times, four times, and five times per fifteen days, respectively. The result implies that the most frequent watering per fifteen days was three times.

Table 1: Socioeconomic characteristics of onion producers (N=205)

**Socioeconomics Variables**		**Mean (%)**	**STD**
Output in (kg)		2965	2958
DAP (kg)		49.8 (195)	75.8
UREA (kg)		34 (179)	24.74
Labor (MDE)		62.61	55.63
Ox (OXD)		7.08	5.06
Plot size (ha)		0.36	0.24
Seed (kg)		2.10	5.00
Livestock holding		8	4
Age (year)		44.3	9.7
Level of education(year)		1.46	1.33
Family size (number)		6	2
Farming experience (year)		5	3.7
Extension frequency of contact		3.4	1.91
Watering frequency per 15days		**Number**	**Percent**
Two times		35	17.07
Three times		100	48.78
Four times		68	33.17
Five times		2	0.98
Training on production	Yes	71	34.63

	No	134	65.37
Training on marketing	Yes	28	13.66
	No	177	86.34
Slope	Plain	163	79.51
	Gentle	42	20.49

Source: Computed from Field Survey Data, 2015/16

3.2. Econometrics Analysis

The maximum likelihood estimation of parameters of the stochastic production frontier model is estimated using the Frontier 4.1 version computer program. Before proceeding to examine the parameter estimates of the production frontier and factors that affect the inefficiency of irrigated onion producers; VIF and CC tests indicated that there were no multicollinearity problems among continuous variables and discrete variables, respectively (see in appendix).

The first null hypothesis is that the check for the existence of the inefficiency part of the composed error term of the Stochastic Frontier Model. This could be created to come to a decision whether or not the traditional average production function (OLS) most closely fits the data set as compared to the stochastic frontier model (SFM) selected for this study. The generalized likelihood-ratio $\{LR = -2[\ln L(H_0) - \ln L(H_1)]\}$ statistic for testing the absence of technical inefficiency impact from the frontier is calculated to be$\{LR = -2 * (-228.39903 + 199.82662) = 57\}$. This value exceeds the critical x^2 $(5\%, 1)$ value of 3.84 at 5% level of significance in Table two. Thus, the null hypothesis was not accepted indicating that the stochastic frontier production function was an ample illustration of the data, given the corresponding ordinary least squares production function. Hence, the stochastic frontier approach most closely fits the data into thought.

The second null hypothesis tested was, test for the selection of the appropriate functional form for data; Cobb-Douglas versus trans-log production function the decision to select the functional form depends on the calculated likelihood ratio. The calculated Log-likelihood Ratio $(LR = -2 * (-199.82662 + 185.31493) = 29.02)$ and the critical value of x^2 at 21 degrees of freedom and 5% significance level is 32.67 in Table 2. Thus, the null hypothesis that all coefficients of the interaction terms in the trans-log specification are equal to zero was accepted. This implies that Cobb-Douglas functional form adequately represents the data under consideration. Hence, Cobb-Douglas functional form was used to estimate the technical efficiency of sample households.

The third null hypothesis explored is that farm-level technical inefficiencies are not affected by farm and socio-economic variables included in the inefficiency model. The inefficiency effect was calculated using the value of the Log-Likelihood function $\{LR = -2[-224.25967 + 199.82662 =$

48.87]}. The calculated LR value of 48.87 was greater than the critical value of 18.31 at 10 degrees of freedom, this shows that the null hypothesis (H_0) that explanatory variables are simultaneously equal to zero was not accepted at 5% significance level. Hence, these variables simultaneously explain sources of efficiency differences among sample households.

Table 2: Summary of the test of hypothesis

Null hypothesis	Degree of freedom	LR	x^2value	Decision
$H_0: \gamma = 0$	1	57	3.84	Not accepted
$H_0: \beta_7 = \cdots = \beta_{27} = 0$	21	29.02	32.67	Accepted
$H_0: \delta_0 = \cdots = \delta_{10}$	10	48.87	18.31	Not accepted

At 5% significance level

Source: Computed from Field Survey Data, 2015/16

3.2.1. Estimation of Cobb-Douglas's production function

Stochastic frontier production function estimates of irrigated onion producers in the North Gondar Zone are presented in Table 3. The value of gamma (γ) is between 0 and 1. If γ is close to zero, it implies that deviations from the production frontier are entirely due to random noise, while a value around unity indicates that most of the deviations are due to inefficiency. Technical efficiency analysis of irrigated onion production revealed that there was a presence of technical inefficiency effects in irrigated onion production in the study area as confirmed by a gamma value of 0.66 that was significant at 1% level. Thus, we can infer that 66% of the deviations from the efficient frontier come from technical inefficiency sources. Or it implies that about 66% variation in output of onion producers was due to differences in their technical efficiencies (total variation in output is due to the existence of production inefficiency). By implication, about 34% of the variation in output among producers is due to random factors such as unfavorable weather, the effect of pests and diseases, errors in data collection and aggregation, and the like. Table 3 showed that coefficients of oxen power, plot size, and DAP had the expected positive signs which indicated that a unit increase in these inputs will lead to an increase in irrigated onion output.

Oxen power days' variable was found to be an important variable for the production of irrigated onion and with an expected sign and statistically significant at 10% significance level. The positive coefficient shows that an increase in the number of oxen-days in course of land preparation activity by 1% will tend to increase onion yield by 0.18%; other variables in the model remain constant. It is the second critical variable, which determines the level of onion production given plot size and DAP. This agrees with the findings of Abate *et al.* (2019) and Abdulkadir (2015).

Onion plot size (ha): The result shows that access to the farm plot is important for explaining the differentiation in the output of each farmer. The elasticity of onion production to farm plots is positive and at 1% level of significance. This implies that an increase in 1% of onion plot size can result in a 0.64% increase in the total output of onion, which onion output is sensitive to plot size. This is consistent as hypothesized and suggests that farmers with larger plot sizes showed significantly higher levels of technical efficiency. It is the first important input that determines onion output. It is the critical variable, which affects the level of onion output given the amount of DAP and oxen days kept constant. Thus, plot size is crucial to increase technical efficiency in onion production in the study areas. The land occupied the highest elasticity of output that indicates the most dominant factor of production. This agrees with the findings of Abebe (2009) and Getahun (2014).

The coefficient of the rate of *DAP fertilizer* is statistically significant at 10% significance level and carries an expected positive sign. This implies a 1% increase in the rate of DAP fertilizer until the recommended rate; onion output will increase by 0.10%. It is the third critical variable, which affects the level of onion output given plot size and oxen days kept constant.

3.2.2.Determinants of technical efficiency

After estimating technical inefficiency variables by using single-stage estimation approach of stochastic frontier model, the significant factors of technical efficiency of onion producers are as follows:

Family size was found to have affected on technical efficiency levels negatively and significantly at 1% level. This was because of the poor managerial ability to effectively utilize the obtainable labor power within the family. This implies that as household size increases, technical efficiency decreases. But this depends fundamentally on two factors, namely; the number of people in a household who can work on the farm and the length of time for which each member are prepared to work on the household farm. Consequently, what matters is not the dimensions of the intrinsically, however the composition and quality of those capable of working on the farm. This agrees with the findings of Matthew and Fatimoh (2008), Kwabena *et al.* (2014), and Musa *et al.* (2014). However, Shumet (2011), Ohajianya *et al.* (2014), Isah *et al*. (2013), and Abebe (2009) had the contrasting result that being household size increases technical efficiency.

Training on marketing contributes negatively and considerably to decrease technical efficiency at 5% level. This could rather be since the training offered is insufficient that is it is not continuous or

it is seasonal training and/ or unrelated that is some concerns might be political. This agrees with the findings of Nyagaka *et al.* (2010) and Wondimu (2013), however it is contrary to the findings by Abebe (2009) and Essa *et al.* (2011).

Education affects positively and significantly to decrease technical efficiency at 10% level. This implies that as the level of education increases, the technical efficiency of onion production increases. The possible reason is the more educated people are well equipped in farm management than the one who takes short-term training. Education helps farmers to incorporate the latest scientific advances and technology tools into their daily operations. So, onion production needed special attention during the production.

Livestock holding: it was affected technical efficiency positively and significantly at 10%. This implies that households who have more livestock holding may not have difficulties purchasing inputs like seed, fertilizer, and the like, and also oxen ownership is among livestock units considered which helps farmers in land preparation. Thus, an increase in livestock holding increases the technical efficiency of onion production. As regards livestock holding, the result in this study is consistent with the findings of other empirical works Beyan *et al.* (2013).

Farming experience: was affected technical efficiency positively and significantly at 5%. It enhances the technical efficiency of households since it increases the farm managerial capacity of households through learning by doing (Ojo M.A *et al. 2009*). Farming experience has been improving the farmer's skill at onion production. A more experienced farmer motivation has a lower level of uncertainty about the innovation's performance. Farmers with higher experience appeared to have often full information and better knowledge and can evaluate the advantage of the innovation considered.

Watering frequency: was affected technical efficiency positively and significantly at 1%. It is important as per the suggested rate. Onions are very sensitive to water stress. Although onions will survive long periods of drought, water handiness is vital for growth and high yields of quality bulbs. Contingent with this, the watering frequency was found to boost technical efficiency through available onion-required water adequately. Additionally, farmers in the study area use the furrow irrigation (flooding) method to irrigate their onion farms.

Table 3: MLE of Parameters of Cobb-Douglas stochastic production Frontier Function for Onion producers

Variable	Parameter	Maximum likelihood estimate	
		Coefficient	t-ratio
Intercept	β_0	2.3	2.9***

LnOx (ODE)	β_1	0.18	1.91*
Lnlabor (MDE)	β_2	-0.06	-0.89
Lnplot size	β_3	0.64	6.4***
LnSeed	β_4	0.05	0.93
LnDAP	β_5	0.10	1.71*
LnUREA	β_6	-0.04	-0.77
Inefficiency effect model			
Constant		0.95	1.63
Age		-0.01	-0.43
Education		-0.14	1.74*
Family size		0.12	2.74***
Livestock holding		-0.05	-1.65*
Experience		-0.08	-2.09**
Extension frequency		0.05	0.20
Slope		0.08	0.34
Training-production		-0.36	-1.17
Training-marketing		0.74	2.09**
Watering frequency		-0.69	-2.77***
Sigma-squared	σ^2	0.60	4.6***
Gamma	Γ	0.66	4.77**
LL		-199.8	
Mean TE		53	
Total sample size	N	205	

***,** Represents significance at 1% and 5% probability levels, respectively

Source: Computed from Field Survey Data, 2015/16

3.2.3. Technical Efficiency Analysis

The maximum likelihood estimates of the Cobb-Douglas stochastic production function coefficients, which are presented in Table 4, are used to predict the technical efficiencies of the sample individual firms. The results of efficiency analysis revealed that the technical efficiency of the smallholder onion household varied from a minimum of 5.5% to a maximum of 87.3% with a mean of 53%. In other words, on average smallholder onion producer households in the study area incur a 47% loss in output due to technical inefficiency. This implies that on average output can be increased by at least 47% while utilizing existing resources and technology if inefficiency factors are fully addressed or more precisely, on average, the output can be expanded by as much as 47% if appropriate measures are taken to improve technical efficiency. The wide variation in technical efficiency estimates is an indication that farmers are still using their resources inefficiently in the production process and there still exist opportunities for improving on their current level of technical efficiency. This result suggests that many households were not utilizing their production

resources efficiently, indicating that they were not getting most output from their given amount of inputs.

Table 4: Frequency Distribution of Technical Efficiency of Onion producers

TE Level	Frequency	Percent
0.05-0.20	16	7.80
0.20-0.40	47	22.93
0.40-0.60	54	26.34
0.60-0.80	78	38.05
≥0.80	10	4.88
Total	205	100
Mean	0.53	
Minimum	0.055	
Maximum	0.873	

Source: Computed from Field Survey Data, 2015/16

To give a better indication of the distribution of the technical efficiencies, a frequency distribution of the predicted technical efficiencies is presented in Figure 1. The frequencies of occurrences of the predicted technical efficiencies in the range indicate that the highest number of households have technical efficiencies between 0.60-0.80. The sample frequency distribution indicates a clustering of technical efficiencies in the region 0.60-0.80 efficiency ranges, representing 38% of the respondents. The findings also reveal that there is a huge gap between the least technically efficient and the most technically efficient farmers in the study areas.

<Figure 1>

3.2.4. Yield gap due to technical inefficiency

Yield gap may be defined as the difference between technically full efficient yield and observed yield. Therefore, the yield gap is the amount that represents fewer yields due to technical inefficiency. From the Stochastic model defined in equation (3), the technical efficiency of the ith household is estimated to be:

$$TE_i = \frac{Y_i}{Y_i^*} = \frac{f(X_i;\beta)\exp(v_i - \mu_i)}{f(X_i;\beta)\exp(v_i)} = \exp(-\mu_i)$$

Then, solving for Y_i^*, the potential yield of each household is represented as:

$$Y_i^* = \frac{Y_i}{TE_i} = f(X_i;\beta)\exp(v_i)$$

Where TE_i =technical efficiency of the ith sample household in onion production

Y_i^* =The frontier/potential output of the ith sample household in onion production, and

Y_i =The actual/observed output of the ith sample household in onion production

Based on the equation above and using the values of the actual onion output obtained and the predicted technical efficiency indices, the potential onion output was estimated for each sample household in onion production on a hectare basis. The mean result is presented in Table 5 below.

Table 5: Onion yield gap due to technical inefficiency

Variable	Min	Max	Mean	Std. Dev.
Actual yield (kg/ha)	200	25,000	2,965	2,958
TE estimates	0.055	0.873	0.53	0.20
Potential/frontier yield (kg/ha)	1,282.3	28,650.8	4,953	3,435
Yield gap/loss (kg/ha)	421.4	7,006.9	1,988	1,075.6

Source: Computed from Field Survey Data, 2015/16

It was observed that the mean technical inefficiency was 47% which caused a 1,988 kg/ha yield gap of onion on the average with the mean value of the actual output and the potential output of 2,965 kg/ha and 4,953 kg/ha, respectively. This shows that sample households in the study area were producing on average 1,988 kg/ha lower onion output than their potential yield.

The mean levels of both the actual and potential output during the production year were 2,965 kg/ha and 4,965 kg/ha, with the standard error of 2,958 and 3,435, respectively. Figure 2 illustrates that under the existing practices there is room to increase onion yield following the best-practiced farms in the study area.

<Figure 2>

4. CONCLUSION AND RECOMMENDATION

This research was conducted to estimate technical efficiency among small-scale irrigated onion-producing farmers and to identify the determinant factors on the technical efficiency of onion producers in the North Gondar Zone. The test result indicates that the traditional average response function is not an adequate representation of the production frontier. The significant proportion of the residual variation in the stochastic production frontier is due to technical inefficiency. This implies that there is room for improvement through better technical efficiency. The estimated Cobb-Douglas stochastic production frontier shows that there is considerable inefficiency among plots in onion production. The mean efficiency level of 0.53 indicates that production can be increased by 47%. There is also a considerable difference in their efficiency level among plots. Therefore if inputs are employed to their utmost potential, there will be sizeable gain from improvement in technical efficiency. Out of six input variables, three input variables which are oxen, plot size, and

DAP positively affected irrigated onion production. The positive coefficient of these factors shows that increased the use of these inputs will raise the production level to a greater amount. The estimated SPF model together with the inefficiency parameters shows TLU, experience, and watering frequency were influenced inefficiency negatively whereas the level of education, family size, and training in marketing increased the level of technical inefficiency. Supported the findings, the subsequent recommendations are forwarded:

Training on the marketing of onion was found to be affecting negatively technical efficiency whereas education affected technical efficiency positively. So, the onion producers should be capacitated through offering continuous scheduled training and education (integrated adult education) at the existing farmers' training centers (FTC) through experts.

Livestock was found to be a positive contribution to onion production. So, livestock intensification should be taken into consideration since there is no possibility for expanding the land at the household level even though plot size has a strong positive effect on onion production.

Watering frequency has a positive significant effect on onion production. Hence, alternative water sources and efficient watering technology should be taken into consideration since in the current irrigation system (specifically in the study area) farmers used an inefficient irrigation system that is furrow irrigation (flooding).

Family size has a negative influence on the technical efficiency of irrigated onion production. Hence, there should be strengthening and re-allocation of existing family planning policy and over-employed labor in other sectors.

Experience in onion production was found to be positive on technical efficiency. Hence, experienced onion producers should be taken as a role model for less experienced onion producers under irrigation.

Abbreviations

CC: Contingency Coefficient, CSA: Central Statistical Authority, DAP: Di Ammonium Phosphate, FTC: Farmers Training Center, Ln: Natural Logarithm, LR: Log-Likelihood Ratio, MDE: Man Day Equivalent, MLE: Maximum Likelihood Estimator, OLS: Ordinary Least Squares, SFM: Stochastic Frontier Model, TE: Technical Efficiency, TLU: Tropical Livestock Unit, and VIF: Variance Inflation Factor.

Acknowledgment: We presented thanks to the University of Gondar for funding and Districts (Dembia, Gondar Zurai, and Takusa) for facilitating data collections and providing data; the farmers who provided the primary data.

Authors' contributions: Tigabu Dagnew Koye, Abebe Dagnew Koye, and Zework Aklilu Amsalu were carried out "Analysis of Technical Efficiency of Irrigated Onion (*Allium cepa* L.) Production in North Gondar Zone of Amhara Regional State, Ethiopia" study participated in proposal development, data collection, and drafted manuscript. All the authors read and approved the final manuscript preparation.

Funding: the University of Gondar was given financial support for the successful completion of this research work

Availability of data and materials: The authors want to declare that they can submit the data at any time based on the publisher's request. The datasets used and/or analyzed during the current study were available from the authors on reasonable request.

Ethical approval and consent to participate: Ethical clearance letters were collected from the University of Gondar Research Coordinator and Chilga Districts (Dembia, Gondar Zurai, and Takusa) of face to care for both the study participants and the researchers. During the survey, official letters were written for each kebele/village, informed verbal consent was obtained from each client, and confidentiality was maintained by giving codes for each respondent rather than recording their name. Study participants were informed that clients have a full right to discontinue or refuse to participate in the study. Hence, all participants throughout the research, including survey households, enumerators, and supervisors were fully informed of the objectives of the study. They were approached friendly in free moods until they do this research.

5. REFERENCES

Abate TM, Dessie AB, Mekie TM. Technical efficiency of smallholder farmers in red pepper production in North Gondar zone Amhara regional state, Ethiopia. *J Econ Struct*. 2019, **https://doi.org/10.1186/s40008-019-0150-6**

Abdulkadir K.O. An evaluation of the efficiency of onion producing farmers in irrigated agriculture: Empirical evidence from Kobo district, Amhara region, Ethiopia; *Int. J. Agric. Extension Rural Dev. 2105,* ISSN: 3254-5428 vol. 2 (5), pp. 116-124

Abebe Dagnew. Technical Efficiency of Onion Production under Irrigation: The Case of Kalu District of South Wollo, Ethiopia. M.Sc. Thesis, Alemaya University. 2019.

Agumas B., Abewa A., Abebe D. Response of Irrigated Onion (*Allium cepa* L.) to Nitrogen and Phosphorus Fertilizers at Ribb and Koga Irrigation Schemes in Amhara Region, Northwestern Ethiopia. *Int. Res. J. Agric. Sci. Soil Sci*. 2014, 4(5):95-100

Aigner D., Lovell C.K., and Schmidt P. Formulation and estimation of stochastic frontier production function models. *Journal of econometrics*; 1977, 6(1): p. 21–37.

Alemayehu Derege. Analysis of Factors Affecting the Technical Efficiency of Coffee Producers in Jimma Zone, Ethiopia. M.Sc. Thesis, Addis Ababa University. 2010.

Alemu MD, Tegegne B, Beshir H. Technical Efficiency in Teff (Eragrostisteff) Production: The Case of Smallholder Farmers in Jamma District, South Wollo Zone, Ethiopia. *Journal of Agricultural Economics and Rural Development;* 2018, 4(2): 513-519.

Aman RD., Bezabih EL., Jema Haji M., & Ketema BD. Vegetable Production Efficiency of Smallholders' Farmer in West Shewa Zone of Oromia National Regional State, Ethiopia. *American International Journal of Agricultural Studies; 2019,* ISSN: 2641-4155 Vol. 2, No. 1

Asfaw DM. Analysis of technical efficiency of smallholder tomato producers in Asaita district, Afar National Regional State, Ethiopia. *PLOS ONE; 2021,* 16(9): e0257366. **https://doi.org/10.1371/journal.pone.0257366**

Bekele Alemayehu. Technical Efficiency Variation for Smallholder Irrigated Maize Producers: A Case Study of Tibila Surface Water Irrigation Scheme, Ethiopia. M.Sc. Thesis, Mekelle University. 2013.

Beyan Ahmed, Jema Haji and Endrias Geta. Analysis of Farm Households' Technical Efficiency in Production of Smallholder Farmers: The Case of Girawa District, Ethiopia. *American-Eurasian J. Agric. & Environ. Sci., 2013,* 13 (12): pp. 1615-1621.

Bravo-Ureta, B.E., and Pinheiro, A.E. Technical, Economic and Allocative Efficiency in Peasant Farming: Evidence from the Dominican Republic. *The Developing Economies*; 1997, 34:48-67

Coelli T.J. and Battese G.E. A model for technical inefficiency effects in a stochastic frontier production function for panel data. *Empirical economics*; 1995. 20(2): p. 325–332.

Coelli TJ. Recent Development in Frontier Modeling and Efficiency Measurement. *Aust. J. Agric. Econ*; 1995, 39: 219-245

Coelli, T. J. A Guide to FRONTIER Version 4.1: A Computer Program for Stochastic Frontier Production and Cost Function Estimation. CEPA Working Paper No. 7/96. Armidale: Department of Econometrics, University of New England; 1996.

CSA (Central Statistical Agency). Agricultural Sample Survey, Report on area and production of major crops, private peasant holding, Meher season statistical bulletin 278, Ethiopian Ministry of Agriculture and Natural Resource, Addis Ababa, Ethiopia; 2015, 1: 22.

Dawit Moges. Resource Use Efficiency in Wheat Production on Small Farms: The Case of Gedeb Hasasa District, West Arsi Zone, Oromia Regional State of Ethiopia. M.Sc. Thesis, Haramaya University. 2012.

Demissie, T., A. Ali and D. Zerfu. Availability and consumption of fruits and vegetables in nine regions of Ethiopia with special emphasis on vitamin A deficiency; *Ethiop J. Health Dev.*; 2009, 23(3):216-222.

Dessale M. Analysis of technical efficiency of smallholder wheat-growing farmers of Jamma district, Ethiopia; *Agriculture & Food Security*; 2019, 8(1): p. 1–8.

EHDA (Ethiopian Horticulture Development Agency). Exporting Fruit and Vegetables from Ethiopia: Assessment of development potentials and investment options in the export-oriented fruit and vegetable sector; 2011.

Essa C. Mussa, Gideon A. Obare, Ayalneh Bogale, and Franklin P. Simtowe. Resource use Efficiency of smallholder Crop Production in the central highlands of Ethiopia; 2011.

Ethiopian Investment Agency. Investment opportunity profile for the production of fruits and vegetables in Ethiopia; 2012.

Fantu Nisrane, Guush Berhane, Sinafikeh Asrat, Gerawork Getachaw, Alemayahu Seyoum Taffesse, and John Hoddinott. Sources of inefficiency and growth in agricultural output in subsistence Agriculture: A stochastic frontier research institute Ethiopia Strategy Support Program II, Ethiopia. ESSPII Working paper 019; 2011.

Fitsum, G, K Woldetsadik and Y Alemayehu. Effect of Irrigation Depth and Nitrogen Levels on Growth and Bulb Yield of Onion (*Allium cepa* L.) at Algae, Central Rift Valley of Ethiopia; 2016.

Gambo, B.A. Effect of Nitrogen, Potassium, Cow Dung and Weed Interference on the Growth and Yield of Onion (*Allium cepa L.*) in Semi-Arid Environment. Unpublished *Ph.D. Thesis*, Department of Crop Science, Usmanu Danfodio University, Sokoto.152p; 2008.

Getahun Mengistu. Technical Efficiency in Onion Production: The Case of Smallholder Farmers in Dugda Woreda, East Shewa Zone, Ethiopia. M.Sc. Thesis, Haramaya University. 2014.

Isah, H., Adebayo, E.F. and Offar Gwandi. The efficiency of Sole Cowpea Production in Gombi Zone of Adamawa State Agricultural Development Program, Nigeria. *American Journal of Advanced Agricultural Research (Ajaar), 2013,* Vol. 1, Issue. 1, pp. 25-41.

Kwabena Nyarko Addai, Victor Owusu, and Gideon Danso-Abbeam. Effects of Farmer – Based-Organization on the Technical Efficiency of Maize Farmers across Various Agro-Ecological Zones of Ghana. *Journal of Economics and Development Studies,* 2014, Vol. 2, No. 1, pp. 141–161.

Leavy, A., Matthews, A., and O'Neill, S. Farm Technical Efficiency and Extension. Economic Papers 9912, Dublin: Economics Department, Trinity College, 1999.

Matthew, O., Adewumi and Fatimoh, A., Adebayo. Profitability and Technical Efficiency of Sweet Potato Production in Nigeria. *Journal of Rural Development, 2008,* 31(5): pp 105-120.

Meeusen W. and van Den Broeck J. Efficiency estimation from Cobb-Douglas production functions with composed error. *International economic review*; 1977, 18(2): p. 435–444.

Mekonnen E., Geta E., and Legesse B. Production efficiency of sesame in Selamago district of south Omo zone, southern Ethiopia. *Current Research in Agricultural Sciences*; 2015, 2(1): p. 8–21.

Melisew M. Organization & management of irrigation schemes in Eastern Amhara, Ethiopia: In the case of Sanka traditional & Golina modern irrigation schemes. Project Paper Presented to the Faculty of the Graduate School of Cornell University in Partial Fulfillment of the Requirements for the Degree of MPS in Integrated Watershed Management and Hydrology. 2012. **http://soilandwater.bee.cornell.edu/publications/Melisew_MPS_2012.pdf**.

Morais, G.A.S.; Silva, F.F.; Freitas, C.O.d.; Braga, M.J. Irrigation, Technical Efficiency, and Farm Size: The Case of Brazil. *Sustainability; 2021, 13,* 1132. **https://doi.org/10.3390/su13031132**

Musa, H., Ahmed, Lemma, Z. and Endrias, G. Technical Efficiency of Maize Producing Farmers In Arsi Negelle, Central Rift Valley of Ethiopia: Stochastic Frontier Approach. *Agriculture and Forestry,* 2014, Vol. 60 Issue 1: 157-167.

Nyagaka, D.O., Obare, G.A., Omiti, J.M., Nguyo, W. Technical Efficiency in Resource Use: Evidence from Smallholder Irish Potato Farmers in Nyandarua North District, Kenya. *African Journal of Agricultural Research;* 2010, 5(11), 1179-1186.

Ohajianya, Otitolaiye, Saliu, and Ibitoye. Technical Efficiency of Sweet Potato Farmers in Okene Local Government Area of Kogi State, Nigeria. *Asian Journal of Agricultural Extension, Economics & Sociology;* 2014, 3(2): pp. 108-117.

Ojo M.A., Mohammed U. S., Adeniji B., and Ojo A.O. Profitability and Technical Efficiency in Irrigated Onion Production under Middle Rima Valley Irrigation Project in Goronyo, Sokoto State Nigeria. *Continental J. Agricultural Science;* 2009, 3: 7 – 14.

Raman, A. M. Vegetable Crops. Drought Advisory. EM4830E: 1 – 2; 2011.

Shumet Asefa (2011) Analysis of technical efficiency of crop-producing smallholder farmers in Tigray, Ethiopia. M.Sc. Thesis, Mekelle University. 2011.

Tabor, G. and M. Yesuf. Mapping the current knowledge of carrot cultivation in Ethiopia, paper submitted to Carrot Aid, Denmark.2012.

USDA (United States Department of Agriculture). United States Department of Agriculture, Onion Production Data; 2009. **http://www.alligationrivergroves.com**

Wassihun AG, Koye TD and Koye AD. Analysis of technical efficiency of potato (*Solanum tuberosum* L.) production in Chilga District, Amhara National Regional State, Ethiopia. *J Econ Struct*., 2019. **https://doi.org/10.1186/s40008-019-0166-y**

Wilson, P. and Hadley, D. Measuring and Explaining Technical Efficiency in UK Potato Production. *Journal of Agricultural Economics*, 1998, 49(3):294-305.

Wondimu Tesfaye. Determinants of Technical Efficiency in Maize Production: The Case of Smallholder Farmers in Dhidhessa District of Illubabor Zone, Ethiopia M.Sc. Thesis, Alemaya University. 2013.

Yemane K, Fasigaw E, Alemat E. Enhancing onion production and productivity through the introduction of seed production techniques in the Central Zone of Tigray Region, Ethiopia. *Academic Journal of Agricultural Research*, 2016, 4: 188-192.

Appendix Table 1: VIF of Explanatory Variables of Stochastic Frontier Production Function Model

Variables	VIF	$^1/_{VIF}$
LnDAP	1.76	0.57
LnODE	1.67	0.60
Lnplotsize	1.56	0.64

LnUrea	1.50	0.67
LnMDE	1.43	0.70
LnSEED	1.28	0.78
Mean VIF	1.53	

Source: Computed from Field Survey Data, 2015/16

Appendix Table 2: VIF for continuous variables used to technical inefficiency model (n=205)

Variables	VIF	1/VIF
Famsze	1.37	0.73
Extfrqnt	1.33	0.75
Age	1.33	0.75
Educ	1.24	0.81
Waterfrnq	1.22	0.82
TLU	1.12	0.89
Exp	1.09	0.92
Mean VIF	1.24	

Source: Computed from Field Survey Data, 2015/16

Appendix Table 3: Contingency coefficients for hypothesized discrete explanatory variables (n=205)

	Trngp	Trngm	Slope
Trngp	1	0.459	0.065
Trngm		1	0.096
Slope			1

Source: Computed from Field Survey Data, 2015/19

Appendix Table 4: MLE of Parameters of trans-log stochastic production Frontier Function for onion Producers

**Variables**	**Coefficient**	**standard-error**	**t-ratio**
Constant	-10.63	6.98	-1.52
LnODE	-0.29	1.31	-0.22
LnMDE	1.05	0.96	1.10
LnSD	0.97	0.91	1.06
LnDAP	1.83	1.07	1.70
LnUrea	0.42	0.98	0.43
LnPlot	1.57	1.60	0.98
$LnODE^2$	-0.29	0.12	-2.31
$LnMDE^2$	-0.01	0.07	-0.18
$LnSD^2$	-0.02	0.04	-0.48
$LnDAP^2$	0.02	0.05	0.35
$LnUrea^2$	-0.04	0.04	-0.96
$LnPLot^2$	0.16	0.13	1.27
LnODE*LnMDE	-0.01	0.13	-0.11
LnODE*LnSD	-0.08	0.12	-0.63
LnODE*LnDAP	0.33	0.12	2.78
LnODE*LnUrea	-0.16	0.09	-1.82
LnODE*LnPlot	0.31	0.17	1.88
LnMDE*LnSD	0.16	0.08	1.97

LnMDE*LnDAP	0.04	0.10	0.35
LnMDE*LnUrea	0.05	0.09	0.62
LnMDE*LnPlot	-0.30	0.13	-2.36
LnSD*LnDAP	0.08	0.07	1.13
LnSD*LnUrea	-0.05	0.07	-0.68
LnSD*LnPlot	-0.18	0.10	-1.79
LnDAP*LnUrea	0.03	0.07	0.36
LnDAP*LnPlot	-0.48	0.15	-3.18
lnUrea*LnPlot	0.03	0.11	0.29
**Efficiency factors**			
Constant	0.17	1.80	0.10
Age	0.18	0.50	0.37
Educ	0.16	0.09	1.94
FAM	0.10	0.05	2.03
TLU	-0.05	0.03	-1.77
EXP	-0.09	0.04	-2.29
EXTF	0.14	0.26	0.52
SLOPE	0.04	0.29	0.15
TRGP	-0.57	0.30	-1.87
TRGM	0.82	0.36	2.25
WTRF	-0.97	0.29	-3.32
**Variance parameters**			
Sigma-squared (σ^2)	0.65	0.16	3.98
Gamma(γ)	0.81	0.09	9.42
log-likelihood function	-185.31		
LR	63.59		
Total sample size	205		

Source: Computed from Field Survey Data, 2015/16

Figure 1: Frequency distribution of technical efficiency

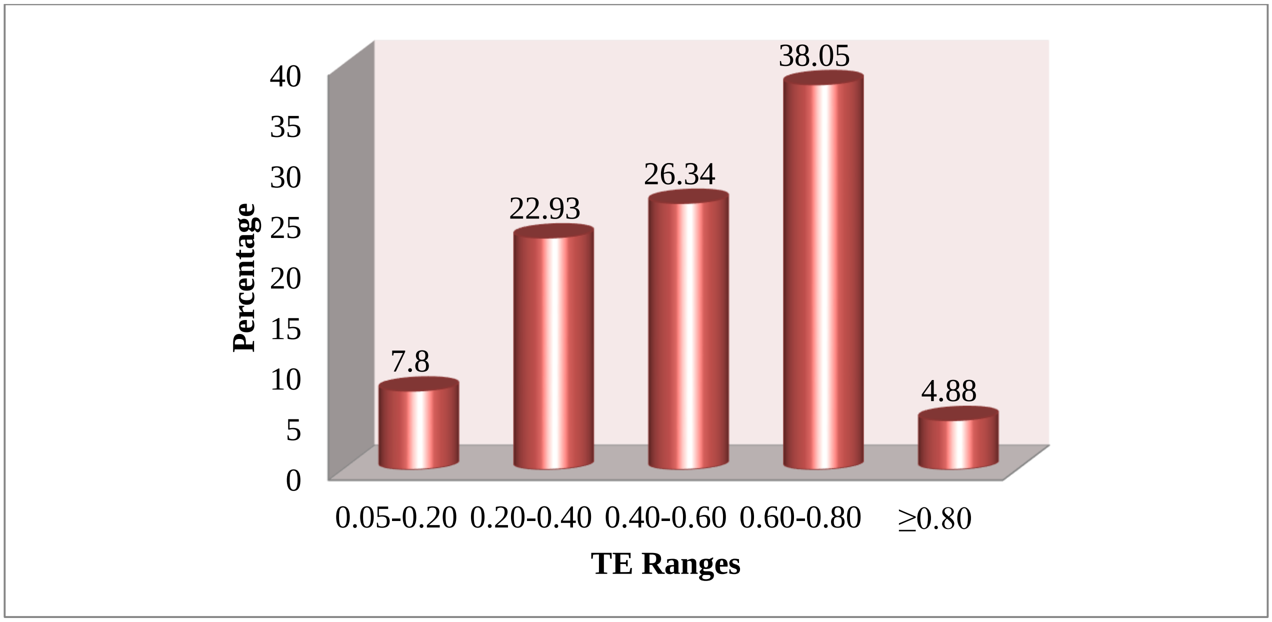

Source: Computed from Field Survey Data, 2015/16

Figure 2: Comparison of the actual and the potential level of yield

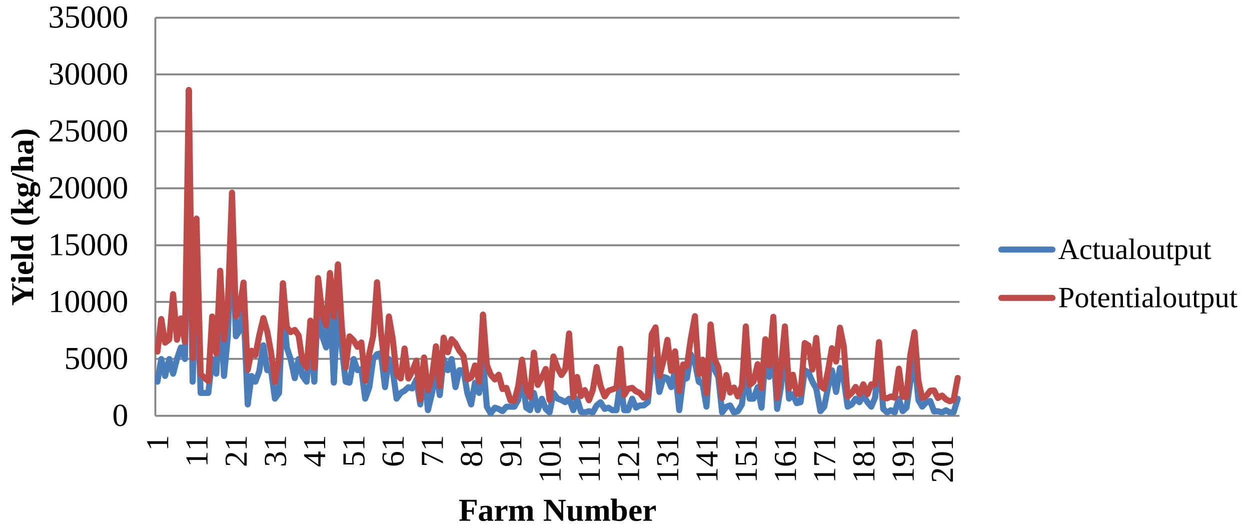

Source: Computed from Field Survey Data, 2015/16
