# Supplementary material for: Analysis of technical efficiency of irrigated onion (Allium cepa L.) production in North Gondar Zone of amhara regional state, Ethiopia
Source: PLoS One. 2022 Oct 13;17(10):e0275177. doi: 10.1371/journal.pone.0275177 (PMC9562163; doi:10.1371/journal.pone.0275177)
Supplement: Attachment — Submitted filename: Response to Reviewers.docx [file pone.0275177.s005.docx]

**Authors’ response**

**For the academic editor**

Dear editors: I have tried to revise it based on the given comments. So, please see the revised manuscript.

The following references are the missing or new citation as the editors’ comment.

Bezabih Emana, Amsalu Ayana, Tesfaye Balemi and Milkessa Temesgen. Scoping Study on Vegetables Seed Systems and Policy in Ethiopia. 2014.

Bornhofen, E., Ramires, T.G., Bergonci, T., Nakamura, L.R., & Righetto, A.J. Associations between global indices of risk management and agricultural development. *Agricultural Systems*, 2019, 173, 281–288. doi:10.1016/j. agsy.2019.03.006

Dagmawe Menelek Asfaw and Abdurhman Kedir Ali. Review on Economic Efficiency of Vegetable Production in Ethiopia. *International Advances,* 2022, Vol. 3, No. 1, pp. 16-24. doi: 10.11648/j.advances.20220301.14

Dawit Soge Soto. Research and Community Services; Annual Report of 2007 E.C Fiscal Year. Wolaita Sodo, Ethiopia. 2014/15.

Demisie R and Tolessa K. Growth and Bulb Yield of Onion (*Allium cepa* L.) in Response to Plant Density and Variety in Jimma, South Western Ethiopia. *Adv Crop Sci Tech*, 2018, 6: 357. doi:10.4172/2329-8863.1000357

Fabrice D, Yann M and Kémal B. Onion (*Allium Cepa*) Production in Urban and Peri-Urban Areas: Financial Performance and Importance of This Activity for Market Gardeners in Southern Benin. *Curr Inves Agri Curr Res*, 2018, 3(2)-. CIACR.MS.ID.000159.

Girma Masresha. Effect of Process conditions on Osmotic Dehydration of *Adama Red* Onion Slice Production. M.Sc. Thesis, Addis Ababa University. 2015.

Habtamu Deribe and Adugnaw Mintesnot. Review on Contribution of Fruits and Vegetables on Food Security in Ethiopia. *Journal of Biology, Agriculture and Healthcare*, 2016, ISSN 2224-3208 (Paper) ISSN 2225-093X (Online) Vol.6, No.11, [www.iiste.org](http://www.iiste.org)

Hanci, F. A comprehensive overview of onion production: Worldwide and Turkey. *Journal of Agriculture and Veterinary Science*, 2018, 11, 17–27.

Jemil Yasin. Economic Efficiency of Onion Producers: The Case of Small Scale Farmers in Kelafo District, Somali Regional State, Ethiopia. *International Researchers,* 2020, Volume No.9 Issue No.1

Przygocka-Cyna, K., Barłóg, P., Grzebisz, W., and Spiżewski, T. Onion (Allium cepa L.) yield and growth dynamics response to in-season patterns of nitrogen and sulfur uptake. *Agronomy*, 2020, 10, 1146. Doi: 10.3390/agronomy10081146.

World Food and Agriculture – Statistical Yearbook. World Food and Agriculture – Statistical Yearbook. 10.4060/CB4477EN. 2021.

Authors’ response

For Reviewer 1:

**IN ABSTRACT PART**

“Onion plays an important role in contributing to a household’s food security and income. But, limited improved seeds, low fertilizers application, low marketing infrastructure, weak institutional support, and market access have contributed to onion’s low productivity”. This problem is a regional issue.

Authors’ response: It will be specify the problems for onion production i.e. “Onion plays an important role in contributing to a household’s food security and income. Despite its importance in the human diet, economic profit and its increasing area coverage, the productivity of onion in the country is much lower on average value.”

Show the full name of DAP?

Authors’ response: Di Ammonium Phosphate

**INTRODUCTION PART**

The recent data need to be shown (in paragraph one)

Authors’ response: it will be changed based on the recent data “Onion (Allium Cepa) is a vital vegetable crop that’s a complimentary product to tomatoes and is of major commercial importance throughout the planet (Przygocka-Cyna *et al.,* 2020). Worldwide production of onion in the year 2019 was 99,968,016 Mg which makes it second to tomato and accounted for 9% of the total share of vegetables (‘World Food Agric. – Stat. Yearb. 2021,’ 2021). China is the leading onion producer with 23,907,509 tons, followed by India 19,415,425 Mg, Egypt 3,115,482 Mg, United States of America 3,025,700 Mg, Iran 2,345,768 Mg and Turkey 2,120,581 Mg (Bornhofen et al., 2019; Hanci, 2018)”.

What’s the hypothesis of this paper?

Authors’ response: the working hypothesis (null hypothesis) sated as follows:

- Smallholder farmers are not technically efficient in irrigated onion production in the study area
- The socioeconomic variables do not significantly influence technical inefficiency

MATERIALS AND METHODS

Description of the Study Area: it is better to present a map of study area

Authors’ response: According to the reviewer’s comment, we have built the map of the study area. Therefore, the map is put in the revised manuscript i. e. on figure 1.

RESULTS AND DISCUSSIONS

Descriptive analysis: what’s the unit of this value (the standard deviation?)

Authors’ response: the standard deviation is unit-less
